# Point-of-care ultrasound detection of catheter-related tricuspid valve infective endocarditis in a patient undergoing haemodialysis

**DOI:** 10.11604/pamj.2026.53.66.51113

**Published:** 2026-02-06

**Authors:** Evans Nii Ayitey MacCready, Isaac Nene Keteku

**Affiliations:** 1 Department of Internal Medicine, Cape Coast Teaching Hospital, Cape Coast, Ghana

**Keywords:** Infective endocarditis, catheter-related infection, point-of-care ultrasound

## Image in medicine

A 32-year-old man with end-stage kidney disease secondary to chronic glomerulonephritis, on maintenance haemodialysis via a right internal jugular central venous catheter for three months, presented with a one-day history of worsening dyspnoea, fever, chills, non-productive cough, and central chest pain. On examination, he was febrile with a temperature of 38.6°C, tachycardic at 126 beats per minute, and his blood pressure was 150/98 mmHg. His oxygen saturation was 88% on room air, which improved to 96% with supplemental oxygen. Chest examination findings were consistent with bilateral pleural effusions. Jugular venous pressure was not elevated, and no murmurs were heard. Point-of-care ultrasound demonstrated a large echogenic mass attached to the anterior leaflet of the tricuspid valve consistent with vegetation (red arrow), with the tip of the central venous catheter visualized within the right atrium (yellow arrow). Mild pericardial effusion and bilateral pleural effusions were also noted. Laboratory evaluation revealed marked leukocytosis with a white blood cell count of 28.6 x 10^9^/L and neutrophil predominance, with severe anaemia (haemoglobin 7.2 g/dL). Blood cultures were obtained for suspected infective endocarditis following the ultrasound findings, and empiric intravenous antibiotic therapy was initiated promptly. However, the patient deteriorated rapidly and died the following day. This image highlights the importance of maintaining a high index of suspicion for catheter-related infective endocarditis in patients undergoing haemodialysis. It demonstrates the critical role of point-of-care ultrasound in the early detection of life-threatening cardiac complications associated with central venous catheters.

**Figure 1:**
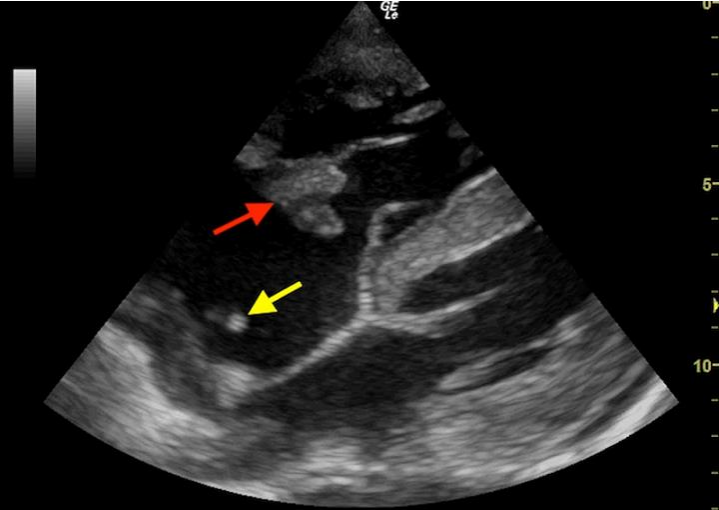
point-of-care ultrasound image demonstrating a tricuspid valve vegetation (red arrow) and the tip of a central venous catheter in the right atrium (yellow arrow)

